# Effect of the Sliding Element Surface Topography on the Oil Film Thickness in EHD Lubrication in Non-Conformal Contact

**DOI:** 10.3390/ma15217549

**Published:** 2022-10-27

**Authors:** Lidia Galda, Jaroslaw Sep, Slawomir Swirad

**Affiliations:** Faculty of Mechanical Engineering and Aeronautics, Rzeszow University of Technology, Powstancow Warszawy 12, 35-959 Rzeszow, Poland

**Keywords:** EHD (elastohydrodynamic), film thickness, non-conformal contact, sliding elements, surface topography

## Abstract

Under hard operating conditions such as high load, low speed and a lack of a sufficient quantity of lubricant, the sliding pairs could suffer serious damage. One of the methods that improves the tribological performance of sliding elements in hard operating conditions is the appropriate surface creation that keeps lubricating substance in cavities. This article presents the results obtained in experimental investigations of the oil film thickness in lubricated non-conformal contact with a different surface topography of the sliding element. The tests were conducted on a ball-on-disc instrument equipped with colorimetric interferometry. Balls of diameter equaled to 19.05 mm were produced from 100 Cr6 steel. To provide hard operating conditions, the glass disc rotated at small speeds in the range of 0.1–0.2 m/s. The tests were carried out at loads of 20 N and 30 N. As a result, in most cases, the highest minimum and average oil film thickness values were obtained when the surface of steel balls was characterized by high negative asymmetry with mainly shallow cavities and some valleys of great depth compared to the height of the peaks. The modified sliding surface that had better performance comprised a comparatively small number of peaks and the curvature of the peaks were large.

## 1. Introduction

The surface roughness of the co-acting elements is a very important factor that significantly influences their performance [1]. The safe operation of sliding elements requires the separation of co-acting surfaces even under hard lubrication conditions. In practice, the mixed lubrication occurs in situations such as start-up or lubricant shortage, but also because of elastic deformation of the co-acting elements during a steady-state operation. Furthermore, the trend to minimize the energy losses leads to setting the machine operation at a possible low friction, but at the same time it is riskier, because of possible oil-film breakdown and metal-to-metal contact [2]. Initially, the contact of the sliding elements is on the local scale, and starting from this stage the dangerous consequences could happen or not. To prevent wear and limit friction of the moving elements, the full film is required that completely separates the co-acting surfaces [3]. In many situations, the film is very thin, and its thickness is smaller than the surface roughness of the co-acting elements. The high pressure inside the contact causes oil compression that leads to a great increase in oil viscosity and elastic deformation of bodies in contact [4,5].

Over last two decades, the texture of the surface became especially important in wear and seizure prevention. The textures, interpreted as the cavities or valleys in a surface, are in specific shape and characterized by a depth, width, coverage area, and are usually in a regular arrangement. There are many examples in which the texture created by different techniques such as a laser process, burnishing or etching brought positive results in tribological characteristics. In the experimental investigation, a more than 50% reduction was obtained in the friction coefficient of the partially textured thrust bearings compared to the untextured ones [6]. The dimensions of the cavities and the area density influence the bearings performance and some results indicated that a great area density of the cavities (40–56%) is beneficial in lowering friction [7,8], but others found that the surface coverage of the cavities should be small [9,10,11,12]. The textures’ dimensions are usually several to several hundred micrometers, but sometimes the cavities are of a nanometer scale. The differences in obtained results and conclusions come mainly from the fact that the research is conducted in dissimilar conditions and the co-acting elements are in different shapes and of different properties. In relation to the surface irregularities, one must admit that they influenced the tribological performance in lubricating contact because the cavities stored some lubricant in case of its shortage. In the elasto-hydrodynamically lubricated (EHL) point contact, Mourier et al. [13] found that deep micro-cavities caused a decrease in the oil film, while shallow micro-pits generated a great increase in the film thickness. The results achieved by Mourier et al. [13] agreed well with the outcomes obtained by Krupka and Hartl [14], who also analyzed the non-conformal lubricated contact of rolling elements. According to their results [13,14] the film thickness depends significantly on the specific depth of the micro-cavity and they suggested that there is a threshold of the cavity depth below which the oil film thickness may increase. Ali et al. [9] obtained a 9% reduction of the friction due to the shallow texture in non-conformal contact of the co-acting surfaces under starved lubrication. Guangteng et al. [15] demonstrated that the surface roughness features influenced the lubricant film distribution in very thin film between rolling elements. According to the results [15], the transverse ridge in the contact increased the mean thickness of the EHD film. Zapletal et al. [16] studied the connection between the film thickness and the friction of uniform surface texture of small irregularities height and of the Gaussian distribution. The authors found that the surface roughness may influence the critical point of the transition from full film to mixed lubrication in non-conformal contact.

In general, it is said that in hydrodynamic lubrication the texture in the form of dimples could disturb the oil film and deteriorate the bearings performance especially in the steady-state condition [17,18]. In such situations the smooth surfaces are recommended, but in fact the so-called smooth surfaces are not perfectly smooth and they have irregularities, of which geometry is very important. The surface topography of the so-called smooth surfaces plays a significant role in the elastohydrodynamic lubrication in non-conformal contact, but locally in slide bearings such contact occurs as in the case of insufficient lubrication or hard operating conditions. If the direct contact of the co-acting elements is often unavoidable, the sliding surface should be prepared suitably. Dobrica et al. conducted a deep study of the roughness orientation of the journal bearing surface on bearing performance in the mixed lubrication regime [19]. The results showed that roughness orientation transverse to sliding direction caused the increase in the film thickness and the decrease in the friction coefficient. The elastic deformation of sliding elements can be dangerous for safe operation, but from the other hand, this deformation can be used to form the full film, because as a result of the high pressure, the oil viscosity increases significantly and enables the surface separation. Looking at the results in EHL non-conformal contact, the sliding surfaces with a small average irregularities height (Sa = 0.0166 µm) obtained a film thickness of 38% higher than the surfaces with greater roughness (Sa = 0.0219 µm) [20]. The modified surface with reduced irregularities height was obtained mainly by the reduction of valleys depth, but also the shape of the irregularities was changed that influenced the reduced parameters of the Sk family which are correlated with the load carrying capacity. Brunetiere and Tournerie [21] analyzed the textured surfaces of mechanical seals that operate under the mixed lubrication regime. They found that the great surface separation had a beneficial effect and the small friction coefficient was due to the interaction between the regular textures (cavities) and the surface roughness beside the texture. These indicated not only the geometry of the cavities, but also irregularities of the whole sliding surface were important to improve tribological performance of sliding elements.

Rosenkranz et al. [22] have made great efforts to classify the recommendation of the texture surface for rolling and sliding elements, and also the specification was done for subgroups such as for seals, gears, bearings and other machine elements. Recently, Song et al. [23] made a detailed review of the texture that was especially devoted to the sliding bearings. Song et al. [23] underlined the effect of the different operating conditions, texture parameters, lubricant properties, geometry, and material of the elements on bearing performance. The metallic elements are the most often examined, but it was also found that sliding bearings with the elastic non-metallic textured element increased the load carrying capacity and decreased the friction due to the specific protrusion [24]. It is also worth noting that most of the theoretical research connected with the properties of EHD film thickness neglects the surface roughness, especially of concrete machine elements, because of the difficulties in the calculation [25] or measurement.

Although much effort has been made to understand the phenomena in lubricated contact of surfaces in theoretical and experimental studies, this research area still requires attention because of its complexity. There are expectations of well-defined morpho-structural characteristics even at the nanometric scale, which are especially welcome for newly designed materials [26] but also for the existing ones.

The aim of this study is to find the surface topography of the sliding element in the non-conformal contact that leads to build the fluid film that assures the great separation of the co-acting surfaces.

## 2. Materials and Methods

### 2.1. Specimens

**Figure 1:**
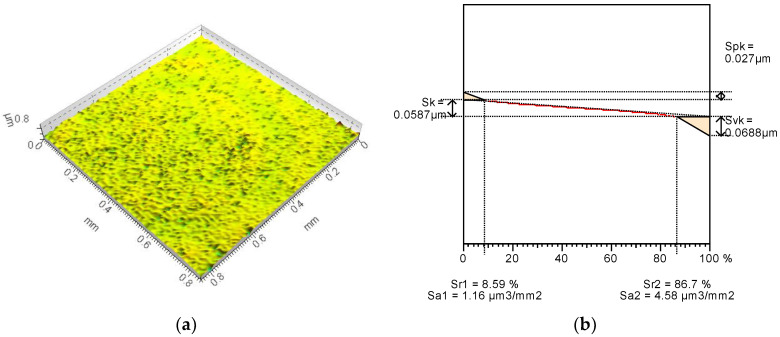
Base surface no. 1 (3D view) (**a**) and Abbott-Firestone curve with Sk-family parameters (**b**) [20].

**Figure 2:**
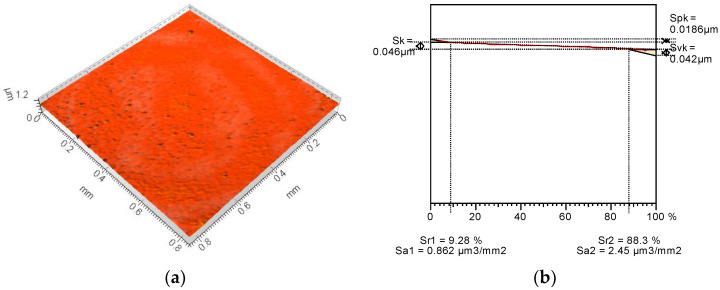
Modified surface no. 2 (3D view) (**a**) and Abbott-Firestone curve with Sk-family parameters (**b**).

**Figure 3:**
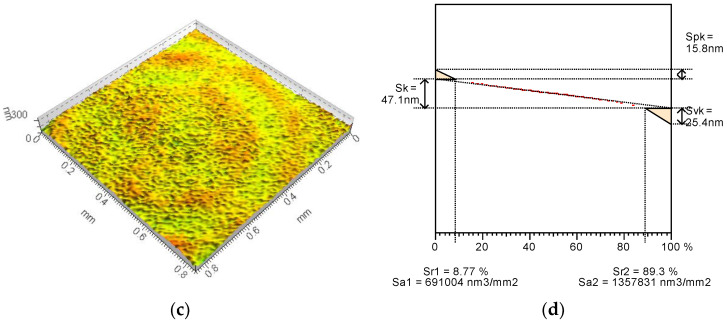
Modified surface no. 3 (3D view) (**a**) and Abbott-Firestone curve with Sk-family parameters (**b**).

To form the surfaces with some reservoirs for the lubricating substance, the polishing process was applied in two different variants. As a reference series (base surface), the factory-new ball surface was tested. The Talysurf CCI Lite white light interferometer was applied for the surface irregularities measurement. The TalyMap Gold (Digital Surf, Besancon, France) application was used to calculate the topography parameters. The base surface (Figure 1) was characterized by fairly low values of Sk-family parameters with a deeper depth of the surface valleys (Svk) than the peaks height (Spk). After the additional polishing process, the values of the reduced height parameters of both modified surfaces decreased. The modified surface no. 2 (Figure 2) contains small peaks and also a surface core of small height. Surface no. 2 comprises comparatively deep depressions but they are shallower than they are in the base surface. Surface no. 2 is the specific one because it contains mainly very shallow cavities but also has some deep depressions. Due to the modification of the surface no. 3 (Figure 3) the reduction in characteristic dimensions presented in the Abbott-Firestone curve was obtained compared to that of base one.

**Figure 4:**
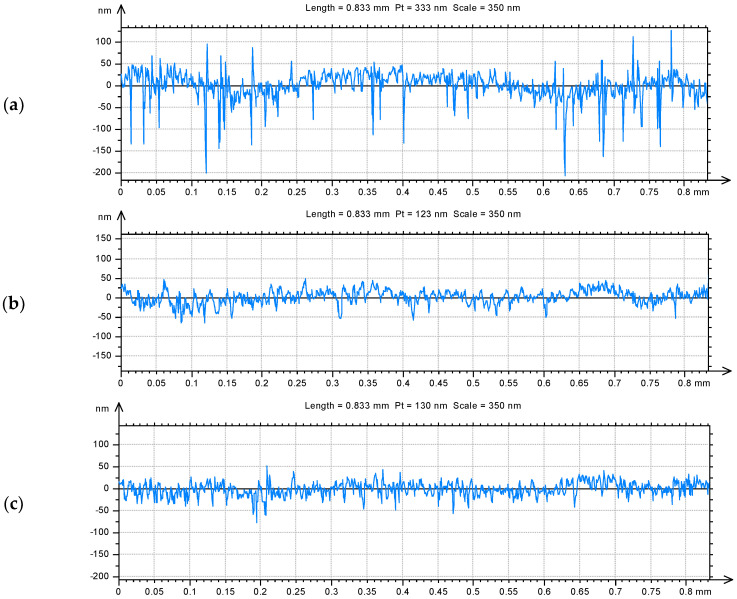
Profiles of surfaces: base no. 1 [20] (**a**), modified no. 2 (**b**) and modified no. 3 (**c**).

Changes in surface irregularities are well seen in the initial profiles of analyzed surfaces (Figure 4). The total height of the profile extruded from the base surface is equal to 333 nm, and after additional polishing process the profiles height values decreased to 123 nm (surface no. 2) and 130 nm (surface no. 3). Peaks of the base surface were greater than 100 nm and the valleys were deeper than 200 nm. The modified surfaces have small peaks up to 50 nm and valleys with a maximum depth of 75 nm. In the exampled profiles, the maximum cavities depth of surface no. 2 is 66.7 nm when surface no. 3 contains valleys of 76.3 nm and the base surface has valleys of 206 nm. Although the modified surfaces no. 2 has some deep depressions, in general contains the shallowest cavities from all examined surfaces. The shape and occurrence frequency of the irregularities are extremely important and the modified surface no. 2 contains less irregularities (PSm = 0.0111 mm) than surface no. 3 (PSm = 0.00843 mm), but the curvature of these irregularities is greater than that of surface no. 3 (Figure 4b,c). The PSm parameter describes an average width between the profile elements (e.g., valleys) of the surface profile.

To describe the surfaces’ geometry, some surface topography parameters from height, spatial and functional groups were selected. Topography parameters were calculated according to the ISO 25178 standard and with special application usage.

The selected surface topography parameters are as follows:

Sq         [µm]                      root mean square roughness

Ssk        -                            skewness

Sku       -                             kurtosis

Sp         [µm]                     maximum peak height

Sv         [µm]                      maximum valley depth

Sz         [µm]                      maximum height

Sa         [µm]                      average roughness

Str        -                             texture aspect ratio

Vv        [mm^3^/mm^2^]          void volume

Vmp    [mm^3^/mm^2^]          peak material volume

Vmc     [mm^3^/mm^2^]          core material volume

Vvv      [mm^3^/mm^2^]          dale void volume

Spd      [1/mm^2^]                 peak density

Spc       [1/mm]                  peak curvature.

In Table 1 the values of selected topography parameters and their changes after an additional polishing process are presented. 

The average roughness Sa of the modified surfaces decreased by over 30% in comparison to the base surface, but the details of the character of the changes were completely different. In case of surface no. 2, the maximum height value increased by 67%, but the Sz value of surface no. 3 decreased by 56% compared to the base surface. The significant increase in the maximum valley depth (135%) of the modified surface no. 2 was achieved, while the Sv of surface no. 3 decreased by 52% in comparison to non-modified sample. It confirms that surface no. 2 was generally smooth, but contains some comparatively deep cavities. Peak heights after additional polishing process were smaller for both surfaces than for the reference one but surface no. 3 contains over two-times smaller peaks than other surfaces. As a result, from the base surface of leptokurtic distribution (Sku = 12.2), new different surfaces were achieved: the surface no. 2 was characterized by a strong leptokurtic distribution (Sku = 177) and surface no. 3 of an almost normal distribution (Sku = 4.27). The asymmetry of all analyzed surfaces was negative, but for modified surface no. 2 a decrease of over two-times was noticed and the Ssk of surface no. 3 was close to 0. The polishing process caused an approximately four-times increase in texture aspect ratio to more than 0.8. Since the average parameters and most of the height parameters decreased, the value of peak and core material volume but also the void volume decreased too. Therefore, the reservoirs of modified surfaces where the lubricating substance may be kept are considerably smaller. The structure of the new surfaces are also completely different. Modified surface no. 2 contains less than 300 peaks on 1 mm^2^ and the peaks curvature is approximately 0.18 mm^−1^ while there are almost 12,000 peaks on 1 mm^2^ and of about 0.05 mm^−1^ of their curvature. The calculated values of the selected topography parameters confirm the observation from the 3D views and the initial profiles.

### 2.2. Test Set

According to EHD theory [27] a film thickness is associated with specific contact conditions and may be presented in the following form:

(1) $$h=k\frac{{\left(U\eta \right)}^{0.7}\alpha^{0.6}E\prime {\;}^{0.03}R\prime {\;}^{0.43}}{w^{0.13}}$$

where: *U*—mean entrainment speed, *η*—viscosity, α—pressure viscosity coefficient, *E’*—effective Young’s modulus, *R*’—effective radius, *w*—load per unit length. The effective radius *R*’ is defined by 1/*R*’ = 1/*R*_1x_ + 1/*R*_2x_ and radii *R*_1x_ and *R*_2x_ are related to two contact elements in the direction of entrainment. Depending on the material of the bodies in contact or other conditions, the results may differ, but what we usually obtain is the central or minimum film thickness. In the case of the eventual influence of surface roughness on the thin film thickness, the research that allows one to obtain the oil film thickness distribution may help to find the correlation or mechanisms of these factors. There are some methods that allow to observe and/or measure the thin film thickness (even of soft materials) such as an optical interferometry, a magnetic resistance, or a laser-induced fluorescence and all of them have their own limitations. To assess the film thickness of the tested contacts, the EHD2 System that is based on the optical interferometry was used. The maximum value of the film thickness, that is an average value of the contact, is up to 1000 nm, and using this device the film thickness distribution of the whole area of the contact may be obtained, but the limit of the film thickness is up to 250 nm. The accuracy of the film thickness is of ±1 nm. The oil film thickness distribution in the non-conformal contact between a stationary steel ball and a rotating glass disc was measured during the tests. The balls of diameter equal to 19.05 mm were made from carbon chrome steel. The hardness of the balls was approximately 61 HRC. The diameter of the glass disc was 100 mm and its thickness was equal to 10 mm. The glass disc was covered with the semitransparent chromium coating. The mechanical properties of the balls and discs are presented in Table 2.

**Figure 5:**
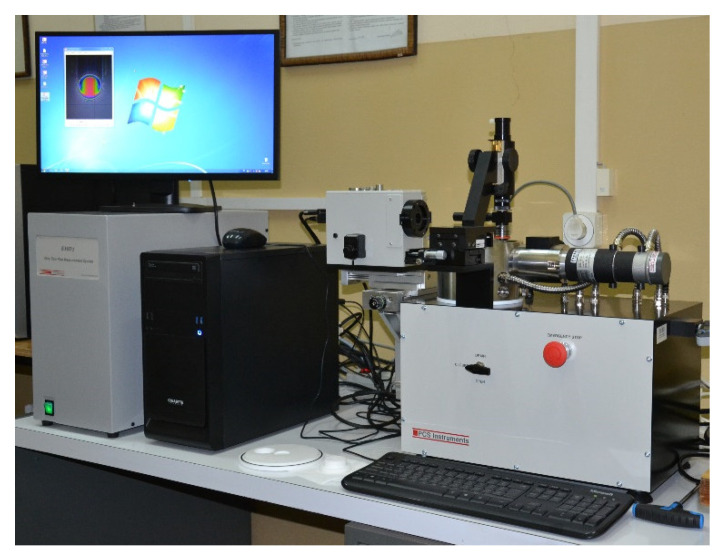
Photo of the EHD2 System for oil film thickness measurement.

The EHD2 System for film thickness measurement with the ball-on-disc sliding pair was equipped with the microscope and CCD camera of a high resolution (Figure 5). The color-space calibration was made for each sliding pair with the special software usage. The CCD camera detected individual R, G, and B values at each pixel and then they were normalized by computer software being the equipment of the rig. The normalizing procedure is done to reduce errors that may be caused by a light source degradation over time [28]. The measurement system of the instrument enables us to grab images of the contact with the color illumination during the disc rotation. The color-space was applied to match the colors in the images to the oil film thickness values in the contact zone.

**Figure 6:**
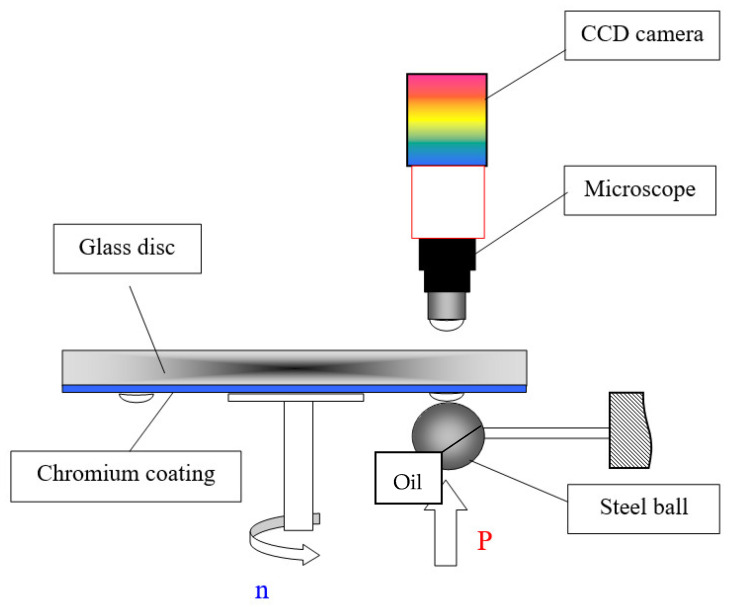
Scheme of the friction pair ball-on-disc.

The stationary ball was pressed against the rotating disc (Figure 6) with the load of 20 N and 30 N. The sliding velocity during the test was in the range of 0.1 to 0.2 m/s. The sliding pair was lubricated with CAS 8042-47-5 mineral oil at the ambient temperature.

## 3. Results and Discussion

**Figure 7:**
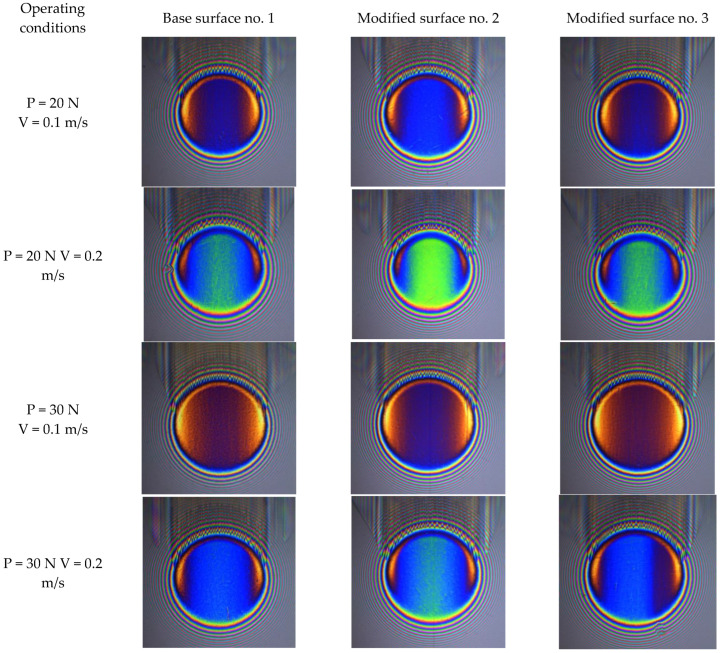
Images of the oil film formed between glass discs and steel balls under different operating conditions.

Figure 7 presents selected images of the oil film formed between the glass disc and the steel ball with different surface topography and with different operating conditions. Although the images give only qualitative assessment of the oil film thickness, the differences are clearly visible. The significant difference in the oil film between glass and disc is observed for differential sliding velocity and also when the load changes from 20 N to 30 N. At the same operating conditions but with different surface finish of the ball surface the differences are perceptible too. The images of film thickness when the ball surface was after modification no. 2 is evident, unlike in comparison to the film of base and modified surface no. 3. In the center of the contact at 20 N and 0.1 m/s the light blue color is in the image, at higher speed of 0.2 m/s mainly light green is seen and at the highest load 30 N and highest speed 0.2 m/s there is green with light blue, while in other series the oil film looks different. The color calibration enables the right values of the oil film thickness calculation.

**Figure 8:**
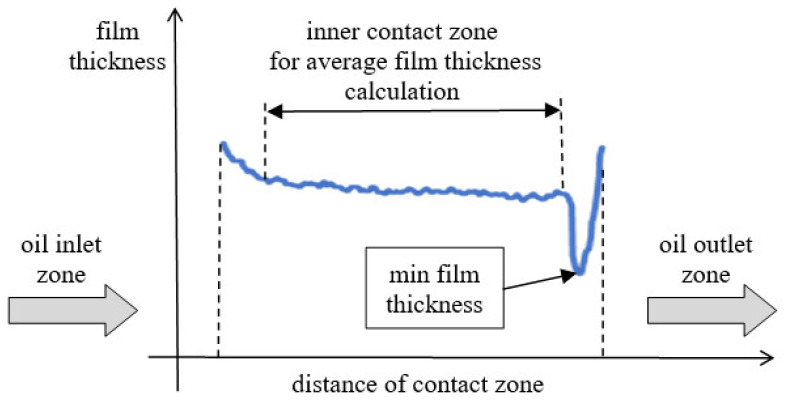
Scheme of the oil film thickness distribution in the non-conformal sliding contact in parallel to the direction of disc rotation.

To assess in detail the oil film thickness of the lubrication gap, two different measurement values of each sliding contact were compared. The first one was the minimum film thickness, because it is the most crucial parameter when one considers how to avoid the direct contact of the co-acting elements. This is especially important in the case of the thin film thickness, when its value is about the height of the surface roughness. In the case of the applied sliding elements in the conducted experiments, the average film thickness from the inner (middle) contact zone was also analyzed. In Figure 8, the scheme of the oil film thickness distribution is presented and on the left of the oil film thickness distribution graph is the inlet zone to the lubrication gap in the contact of examined sliding elements.

In the experiment, the specific counter-specimen in the form of a glass disc must be used to obtain the film thickness distribution. The glass disc is characterized by significantly smaller mechanical properties, and this element is the moving element in the experiment, but although this configuration is rather atypical in industrial application, it allows us to obtain the distribution of thin film thickness. The minimum film thickness of the lubrication gap occurs in the outlet zone with the peak of the fluid pressure profile, and in the oil inlet zone, the film thickness is the maximum. The deformation observed in the experiment may be greater than it is in most of the real sliding elements, because of a specific material application for the disc (glass), but the mechanism is similar.

**Figure 9:**
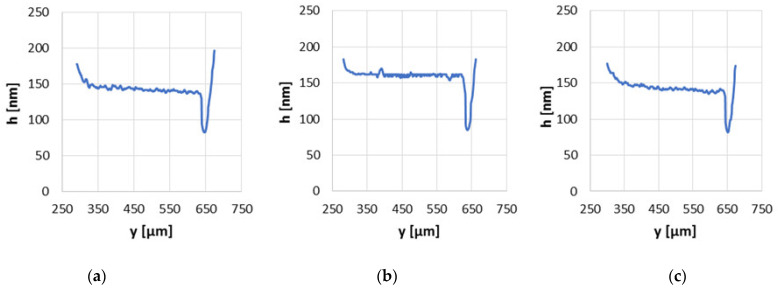
Oil film thickness distribution graphs in non-conformal contact with load of 20 N and sliding velocity of 0.1 m/s for the base surface (**a**), modified surfaces no. 2 (**b**) and no. 3 (**c**).

In Figure 9, the oil film thickness distribution graphs in the non-conformal contact parallel to the direction of slide disc rotation with load of 20 N and speed of 0.1 m/s are presented. The highest minimum and average oil film thickness values were obtained for a contact with the steel surface of modification no. 2. The minimum values of the film thickness were quite similar and those of contact with the modified surface no. 2 was approximately equal to 85 nm whereas the minimum film thickness of contact with the base surface was about 83 nm and 82 nm for modified surface no. 3. The significant difference in the film formed between modified surface no. 2 and the disc in comparison to other series was observed in the middle zone of the contact. The average film thickness of contact with the modified surface no. 2 was more than 160 nm, whereas those surfaces of the base and after modification no. 3 reached approximately 140 nm. Surface no. 2 contains some comparatively deep valleys, but mainly shallow cavities exist on this surface. The number of peaks on surface no. 2 is many times smaller than in other series; 20 times in comparison to base and 43 times in comparison to modified surface no. 3. The curvature of the peaks of the surface no. 2 was relatively large. The differences in the void volume of modified surfaces, where the lubricating substance could be kept, are quite small; this means that the number and shape of the cavities but also of the peaks play the main role in the oil film formation and influence the film thickness.

**Figure 10:**
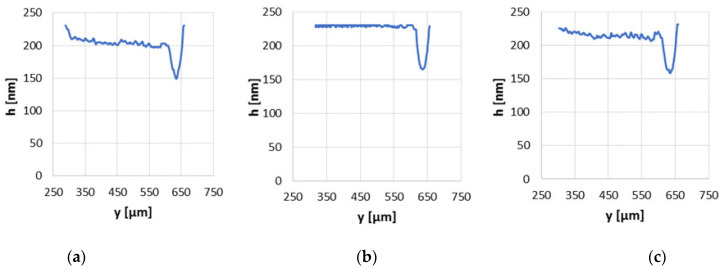
Oil film thickness distribution graphs in non-conformal contact with load of 20 N and sliding velocity of 0.2 m/s for the base surface (**a**), modified surfaces no. 2 (**b**) and no. 3 (**c**).

At the highest sliding velocity from the test range, equal to 0.2 m/s, both the minimum and average values of the oil film thickness differed significantly between series and the highest film thickness values were still observed when the surface no. 2 was examined (Figure 10). The highest minimum film thickness of approximately 165 nm was obtained for a contact with the modified surface no. 2, while with application of the base surface it was less than 150 nm. The supreme average film thickness was equal to almost 230 nm of contact with the modified surface no. 2 and the application of the base surface allows to get the average film thickness of a little more than 200 nm under the same operating conditions. Generally, the values of film thickness increased with the increase in the sliding velocity and this was observed in all analyzed series. The differences in the separation of the co-acting elements of examined series are due to the dissimilar surface irregularities. The modified surface no. 2 with a strong leptokurtic distribution, a negative asymmetry and small peaks of big curvature allowed to obtain the superior results in oil film formation in non-conformal sliding contact. The modified surface no. 3 is characterized by better performance than the base surface, but the values of minimum and average film thickness were smaller than those of the modified surface no. 2. The modified surface no. 3 was characterized by small height parameters, but skewness was close to zero and kurtosis close to 3 (Sku = 4.27), which means that the surface distribution was similar to normal without the predominance of dimples that could accumulate oil, as was in the case of modified surface no. 2.

**Figure 11:**
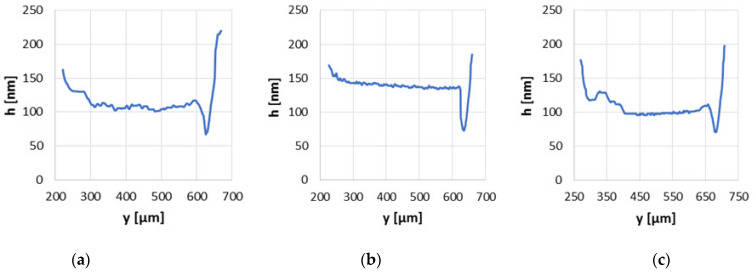
Oil film thickness distribution graphs in non-conformal contact with load of 30 N and sliding velocity of 0.1 m/s for the base surface (**a**), modified surfaces no. 2 (**b**) and no. 3 (**c**).

With the greatest load of 30 N and the smallest velocity of 0.1 m/s the operating conditions became harder due to the large load and small velocity, but in this situation due to the specific shape of the surface topography the average oil film thickness increased the most. For the friction pair with the ball after modification no. 2 the average oil film thickness was more than 27% higher than that of base series (Figure 11). Under those conditions due to the closed cavities in the surface and quite smooth surface around the cavities, the oil was squeezed and because of the high pressure during elastic deformation of non-conformal contact, the oil density could increase significantly and caused greater separation of the elements (h_aver_ = 140 nm). Vladescu [29] obtained a similar percentage increase (28%) in the film thickness test conducted under mixed and boundary conditions with textured surfaces application, but there it was the contact that simulated the piston ring and liner. The base surface and that after modification no. 3 application resulted in the average film thickness of only a few nanometers above 100 nm.

The greatest improvement in the performance and tribological characteristics of sliding elements operating under hard condition, e.g., with a high load and a small speed due to the specific texture application, was obtained by Galda et al. [10,18,30,31] and others [6]. In those studies, authors mainly noticed the smallest friction force or friction coefficient and the operating temperature decrease but also the non-destroyed sliding surfaces that were modified by texture. These observations were found in tests conducted with textured samples in conformal contact. It was difficult to precisely measure the thickness of lubrication gap and such data were usually presented without information about the oil film thickness. The advanced research results of the oil film thickness are presented quite seldom because of some objective difficulties (specific instruments or advanced modelling techniques), but give some good evidence [8,14,21,29,32] that the specific texture may or may not increase the oil film thickness during sliding or sliding/rolling contact under the hard operating conditions. The findings obtained by the oil film thickness measurements also give an explanation to why the friction is smaller in case of a proper texture application in the other similar sliding contacts. Vlădescu et al. [29] presented key conclusions from their research related to the influence of surface texture on friction and film thickness. They found that the 28% increase in film thickness resulted in a 41% decrease in friction under mixed and boundary conditions of lubricated sliding contact due to the specific texture application. In the presented results, the minimum oil film thickness of the contact with the modified surface no. 2 was also superior in comparison to other series, but only by a few nanometers.

**Figure 12:**
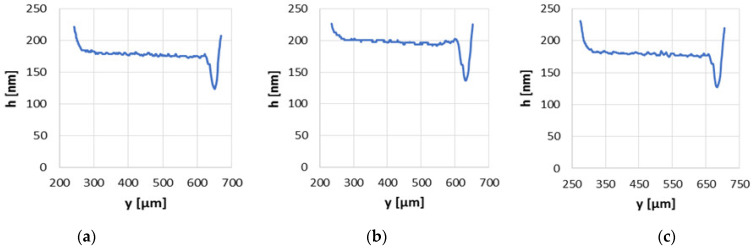
Oil film thickness distribution graphs in non-conformal contact with load of 30 N and sliding velocity of 0.2 m/s for the base surface (**a**), modified surfaces no. 2 (**b**) and no. 3 (**c**).

With the greatest velocity of 0.2 m/s and a load of 30 N, the series of surface finish no. 2 was still the most favorable (Figure 12). The average oil film of modified series no. 2 was approximately 20 nm higher than that of the base series and the minimum oil film thickness was higher more than 14 nm.

**Figure 13:**
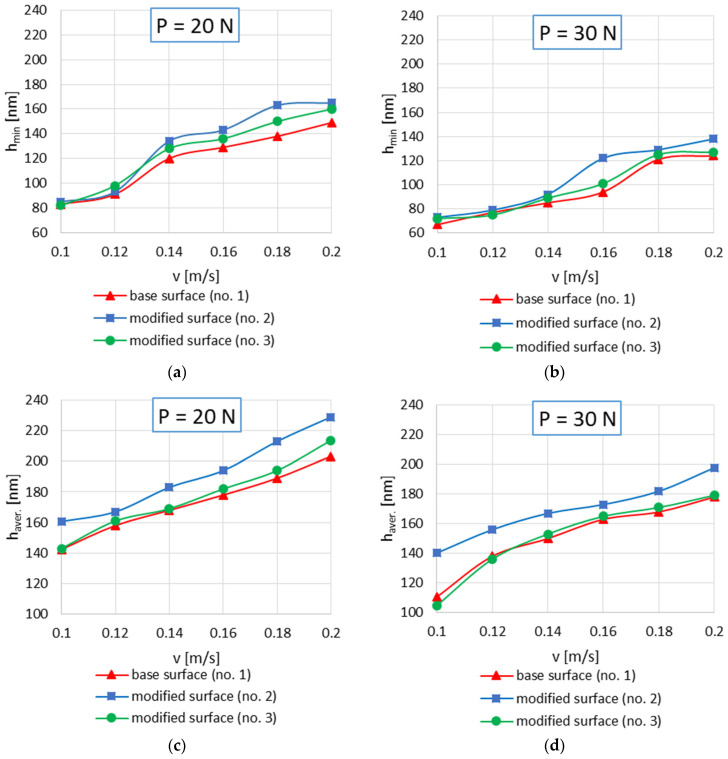
Minimum oil film thickness h_min_ and average oil film thickness h_aver._ values in non-conformal sliding contact with loads of 20 N (**a**,**c**) and 30 N (**b**,**d**).

The surface finish after modification no. 3 resulted in just a few nanometers increase in the minimum and also the average oil film thickness compared to the base variant. With the selected loads and speeds, only modified surface no. 2 with negative skewness and dimples were superior to the other examined series when the minimum and average oil film thickness is taken into account. Considering the other operating conditions in the non-conformal contact of the tested series similar results were found. In Figure 13 the minimum and average values of oil film thickness in the non-conformal sliding contact with loads of 20 N and 30 N and sliding velocity from the range of 0.1 m/s to 0.2 m/s with an interval of 0.02 m/s for all series tested are presented. The film thickness increase with the sliding velocity increase was observed for all analyzed series at the whole range of operating parameters. Generally, higher minimum and average film thickness values were observed when the load was lower P = 20 N than at 30 N. The modified surfaces of smaller average irregularities height and reduced maximum peaks height were superior in effective oil film forming compared to the base surface application. The best performance under hard operating conditions (low speed and high load) was found for sliding surfaces with some comparatively deep cavities but with shallow ones in the majority of contact zone and also with small number of peaks but of the big peaks’ curvature. The surface topography significantly influences the tribological performance in elastohydrodynamic lubrication because the shape, dimensions and frequency of surface asperities (irregularities) have a strong effect on the oil film properties between the sliding elements.

**Figure 14:**
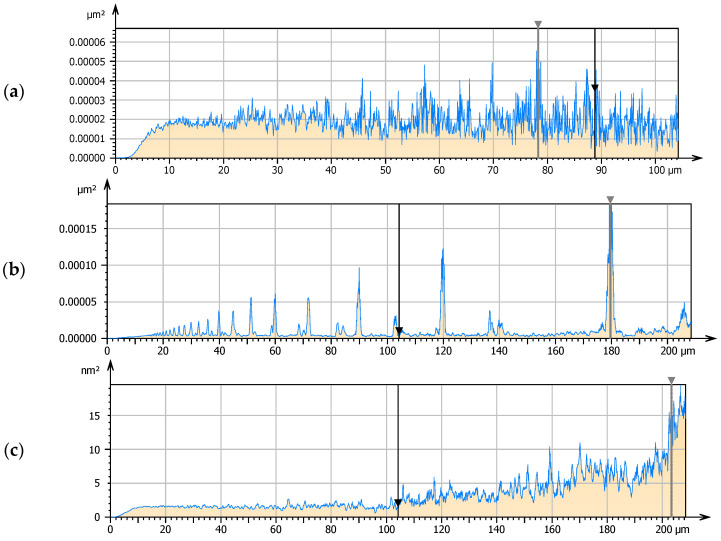
Average power spectral density of surfaces: base no. 1 (**a**), modified no. 2 (**b**) and modified no. 3 (**c**).

The surface texture has been of great interest in the last two decades, and many research results, experimental but also theoretical, were published on this topic. Some authors also indicated that there are results that are confusing, taking into account the optimal texture geometrical parameters. The exact values of the results and also geometry or dimension of the texture created on the superior surface no. 2 presented in this article are far from the geometries characterizing the texture that one may usually find in the literature describing the conformal contact [6,10,18], but what is similar, it is the characteristic of the sliding surface, e.g., leptokurtic distribution, negative skewness, mainly shallow dimples with a few deeper ones and peaks of big curvature. Actually, both examined modified surfaces of completely different surfaces geometry were better than the base surface in film forming and separation of co-acting sliding elements, but the improvement of surface no. 3 application was usually quite symbolic. When one thinks about the specific texture, usually one imagines the regular arrangement of dimples on the surfaces and the typical polishing process is associated with the surface of random distribution of irregularities. In the case of the modified surfaces, the character is not typical for random arrangement of peaks and valleys. The averaged power spectral density graphs of the tested surfaces are completely different (Figure 14).

**Figure 15:**
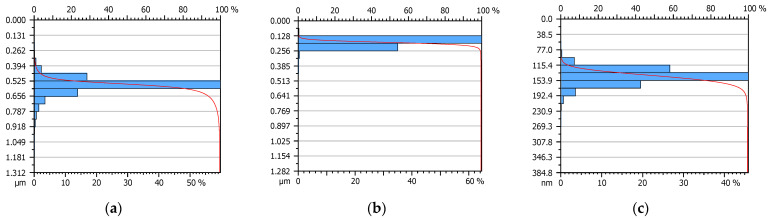
Abbott-Firestone curves of surfaces: base no. 1 (**a**), modified no. 2 (**b**) and modified no. 3 (**c**).

The base surface no. 1 is characterized by a typical random character, which is confirmed by the graphic distribution of the power spectral density (Figure 14a) but modified surfaces, especially surface no. 2, has a mixed character (Figure 14b). It is also hard to say that the surface is of the deterministic distribution because the power spectral density is not representative for such surfaces but there are symptoms that there are asperities that occur in specific frequency and size. These are important features because this surface was characterized by the greatest oil film thickness of all those surfaces tested. Comparing the histograms on the Abbott-Firestone curves of the tested surfaces, the differences in depth and height of irregularities are observed (Figure 15). The frequency of peaks occurrence of surface no. 2 is the smallest of all, and also the depth/height of irregularities is limited.

**Figure 16:**
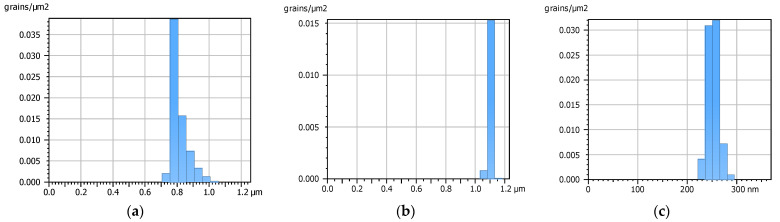
Peak count distribution graphs of surfaces: base no. 1 (**a**), modified no. 2 (**b**) and modified no. 3 (**c**).

Analyzing peak-count histograms, the limited number of peaks size is observed for surface no. 2 and the density of peaks is the smallest for this modified surface (Figure 16) which is also in good correlation with the values of the Spd parameter. It is also important that the peaks of surface no. 2 were in the smallest number and were characterized by the largest peak curvature (Spc parameter).

**Figure 17:**
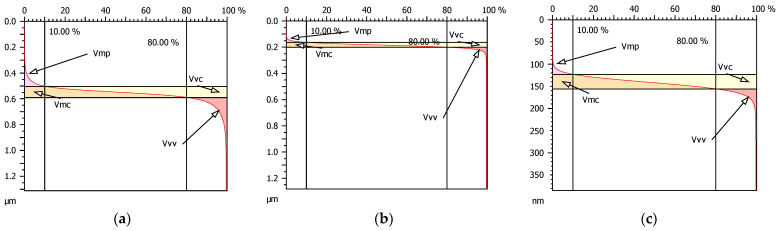
Graphic presentation of volume parameters of surfaces: base no. 1 (**a**), modified no. 2 (**b**) and modified no. 3 (**c**).

Taking into account the size and frequency of the peaks and valleys of the analyzed surfaces, the volume parameters should differ substantially, as is confirmed by graphic representation in Figure 17.

The results obtained are in good qualitative agreement with the results of [9,10,11,12,18,30,31] because the small area of specific cavities (surface no. 2) was the most favorable in the tribological performance enhancement under hard lubrication conditions, but, different in this article, the dimples seemed to be irregularly arranged where very shallow cavities were intermittently occurring with a few deep ones. When analyzing the power spectral density graphs, it was noticed that the character of modified surface no. 2 was not typical for random surfaces. The histograms of the peaks and valleys also highlighted the specific characteristics of surface no. 2 which comprised a small number of rounded peaks and mainly small valleys but with just a few deep pits and this surface was the superior in the greatest film formation. The effects presented in this article are in very good agreement with the results of seals modeling [21] where Brunetiere et al. found that the beneficial effect of surface texture is due to the cavities and the surface surrounding them. The significant factor that influences the best performance of surface no. 2 in the conducted experiment could be the fact that there were just a few deep cavities and very small irregularities around them. Achieved results show that it is difficult to point out one or two surface roughness parameters that influence the most on the film thickness. The design (number of cavities and peaks, their dimensions, geometry, etc.) of the whole surface in the contact is crucial in the better tribological performance enhancement. The results obtained are also in good agreement with some outcomes of the experiments conducted with rolling-sliding non-conformal contact [33,34]. Boidi et al. [33] found a strong correlation between tribological performance and texture depth and according to their results, the shallow textures assured friction reduction. Hansen et al. [34] underlined that not only the height roughness parameters influenced the performance of non-conformal contact, but the shape of asperities played a key role in the increase in load carrying capacity.

## 4. Conclusions

The oil film thickness of non-conformal contact of sliding elements in elastohydrodynamic lubrication under hard operating conditions was measured to assess the influence of the surface topography.

The film thickness of co-acting elements depends on the sliding velocity and the applied load. An increase in the velocity value led to an increase in elements’ separation and the distance between the co-acting elements was greater when the load was lower P = 20 N.

The experimental results show that modified surfaces of small average height and small peaks height but with cavities with differential shape and dimensions are better than the base surface that was brand new of great irregularities height.

The greatest increase of 27% in the average oil film thickness compared to that of base surface application was found when the surface no. 2 was tested with the lowest sliding velocity of 0.1 m/s and the highest load of 30 N. Surface no. 2 was characterized by small average surface irregularities (Sa = 0.0152 µm) but contains some comparatively deep cavities (Sv = 1.1 µm) and the number of peaks was really small Spd = 272 mm^−2^ but their curvature was large Spc = 0.175 mm^−1^. Regarding the obtained results, it may be concluded that on the oil film thickness increase influenced the presence of both some deep cavities as well as comparatively smooth surface (shallow cavities) around them in the majority of the contact zone.

## Figures and Tables

**Table 1:** Values of selected surface topography parameters of the base [20] and modified surfaces and changes of parameter values after modification.

Surface No.		1	2	3	∆ [%] 2 to 1	∆ [%] 3 to 1
Sq	µm	0.0326 ± 2.8%	0.0215	0.0189	−34	−42
Ssk		−1.76 ± 4.0%	−5.01	−0.367	−185	79
Sku		12.2 ± 8.5%	177	4.27	1351	−65
Sp	µm	0.371 ± 9.6%	0.293	0.145	−21	−61
Sv	µm	0.469 ± 4.3%	1.1	0.223	135	−52
Sz	µm	0.839 ± 3.0%	1.4	0.368	67	−56
Sa	µm	0.0219 ± 1.3%	0.0152	0.0147	−31	−33
Str		0.175 ± 20.2%	0.884	0.83	405	374
Vv	mm^3^/mm^2^·10^−5^	3.32 ± 0.9%	2.51	2.4	−24	−28
Vmp	mm^3^/mm^2^·10^−7^	13.5 ± 6.9%	8.63	8.19	−36	−39
Vmc	mm^3^/mm^2^·10^−5^	2.12 ± 1.1%	1.6	1.63	−25	−23
Vvv	mm^3^/mm^2^·10^−6^	5.74 ± 2.4%	2.84	2.44	−51	−57
Spd	mm^−2^	5481 ± 4.1%	272	11869	−95	117
Spc	mm^−1^	0.147 ± 4.4%	0.175	0.0483	19	−67

**Table 2:** Mechanical properties of the ball and disc.

Mechanical Properties		Steel Ball	Glass Disc
E	MPa	2.07·10^5^	7.5·10^4^
v	-	0.293	0.22

## Data Availability

The data presented in this study are available on request from the corresponding author.
